# Incorporating social contact data in spatio-temporal models for infectious disease spread

**DOI:** 10.1093/biostatistics/kxw051

**Published:** 2016-12-24

**Authors:** Sebastian Meyer, Leonhard Held

**Affiliations:** *Institute of Medical Informatics, Biometry, and Epidemiology, Friedrich-Alexander-Universität Erlangen-Nürnberg,Waldstraße 6, DE-91054 Erlangen, Germany*seb.meyer@fau.de; Epidemiology, Biostatistics and Prevention Institute, University of Zurich, Hirschengraben 84, CH-8001 Zürich, Switzerland

**Keywords:** Age-structured contact matrix, Areal count time series, Endemic-epidemic modeling, Infectious disease epidemiology, Norovirus gastroenteritis, Norwalk virus, Spatio-temporal surveillance data

## Abstract

Routine public health surveillance of notifiable infectious diseases gives rise to weekly counts of reported cases—possibly stratified by region and/or age group. We investigate how an age-structured social contact matrix can be incorporated into a spatio-temporal endemic–epidemic model for infectious disease counts. To illustrate the approach, we analyze the spread of norovirus gastroenteritis over six age groups within the 12 districts of Berlin, 2011–2015, using contact data from the POLYMOD study. The proposed age-structured model outperforms alternative scenarios with homogeneous or no mixing between age groups. An extended contact model suggests a power transformation of the survey-based contact matrix toward more within-group transmission.

## 1. INTRODUCTION

The social phenomenon of “like seeks like” produces characteristic contact patterns between subgroups of a population. If suitably quantified, such social mixing behavior can inform models for infectious disease spread (Read *and others*, 2012). One of the largest social contact surveys to date was conducted as part of the EU-funded POLYMOD project, recording conversational contacts of 7 290 individuals in eight European countries (Mossong *and others*, 2008). Contact patterns were found to be similar across the different countries and highly assortative with respect to age, especially for school children and young adults.

The basic idea behind the combination of social contact data with epidemic models has been termed the “social contact hypothesis” (Wallinga *and others*, 2006): The age-specific numbers of potentially infectious contacts are proportional to age-specific numbers of social contacts. For instance, for pathogens transmitted via respiratory droplets, face-to-face conversation and/or physical contact are frequently used as proxy measures for exposure. Many studies have now made use of the POLYMOD contact data (Rohani *and others*, 2010; Goeyvaerts *and others*, 2010, 2015; Birrell *and others*, 2011; Baguelin *and others*, 2013), but none of them accounts for the spatial characteristics of disease spread. The distance of social contacts from the home location of each participant has only recently been investigated by Read *and others* (2014). Their finding that “most were within a kilometer of the participant’s home, while some occurred further than 500 km away” reflects the power-law distance decay of social interaction as determined by human travel behavior (Brockmann *and others*, 2006). Meyer and Held (2014) found such a power law to translate to the spatial spread of infectious diseases.

The purpose of this paper is to combine the social and spatial determinants of infectious disease spread in a multivariate time-series model for public health surveillance data. For notifiable diseases, such data are routinely available as weekly counts of reported cases by administrative district and further stratified by age group or gender. Social contact matrices reflect the amount of mixing between these strata. Our focus is on age-structured models, but the methods equivalently apply to other or multiple strata. We investigate if a (possibly adjusted) contact matrix captures disease spread better than simple assumptions of homogeneous or no mixing between the subgroups. The approach also allows us to estimate how much disease incidence in each group can be linked to previous cases in their own and in other groups—while adjusting for the spatial pattern of disease spread.

This paper is organized as follows. Section 2 introduces our case study on norovirus gastroenteritis, including contact data from the POLYMOD study. Section 3 outlines the spatio-temporal modeling framework and describes how to incorporate additional stratification with a contact matrix. Section 4 shows results of the case study and Section 5 concludes the paper with a discussion. The supplementary material contains additional figures, an animation of the data, as well as the $\texttt{R}$ source package $\texttt{hhh4contacts}$ with the data and code to reproduce the presented analysis (run $\texttt{demo}$ (“$\texttt{hhh4contacts}$”) after installing and loading the package).

## 2. CASE STUDY: NOROVIRUS GASTROENTERITIS IN BERLIN, 2011–2015

Most of the aforementioned studies relate contact patterns to the spread of influenza, whereas here we investigate the occurrence of norovirus-associated acute gastroenteritis. Both diseases are highly infectious, have a similar temporal pattern, and similar mortality in elderly persons (van Asten *and others*, 2012). However, in contrast to influenza, vaccines against noroviruses have yet to be developed (Pringle *and others*, 2015). Absence of vaccination simplifies the analysis of infectious disease occurrence since vaccination coverage—potentially varying across age groups, regions and over time—needs not to be taken into account.

### 2.1. Epidemiology of norovirus gastroenteritis

Norovirus-associated acute gastroenteritis is characterized by “sudden onset of vomiting, diarrhea, and abdominal cramps lasting 2–3 days” (Pringle *and others*, 2015). O’Dea *and others* (2014) estimate an average symptomatic period of 3.35 days from outbreaks in hospitals and long-term care facilities, where vulnerable individuals live closely together and norovirus outbreaks most commonly occur. Another frequently affected subgroup are children in daycare centers. Norovirus incidence peaks during winter, where outbreaks in childcare facilities were observed to precede those in private households, hospitals, and nursing homes (Bernard *and others*, 2014b).

Noroviruses are highly contagious since only few viral particles are needed for an infection. Being thermally stable and particularly persistent in the environment (Marshall and Bruggink, 2011), noroviruses can also be transmitted indirectly via contaminated surfaces or food. The serial interval, i.e. the time between onset of symptoms in a primary and a secondary case, ranges from within a day to more than 1 week with a median of about 3 days (Götz *and others*, 2001).

### 2.2. Incidence data

In Germany, the national public health institute (the Robert Koch Institute, RKI) provides access to incidence data of notifiable diseases through the *SurvStat@RKI 2.0* online service (http://survstat.rki.de). Since the last revision of the case definition for norovirus gastroenteritis in 2011, only laboratory-confirmed cases are reported to the RKI. The number of cases to be modeled thus excludes all asymptomatic cases as well as all those symptomatic cases, who have not found their way to laboratory testing (Gibbons *and others*, 2014). It is known that under-reporting of norovirus illness is most pronounced in the 20- to 29-year-old persons and substantially lower in persons aged $<10$ years and 70 years and over (Bernard *and others*, 2014c). A sensitivity analysis will indicate how under-reporting may affect the interpretation of our model results.

As to the geographic region of interest, we chose the largest city of Germany, Berlin, which is divided into 12 administrative districts. This enables the analysis of disease spread on a smaller spatial scale. Furthermore, a large underlying population is required for our time-series model to be a reasonable approximation of the epidemic process (Farrington *and others*, 2003).

We have downloaded weekly numbers of reported cases of norovirus gastroenteritis in Berlin from *SurvStat@RKI* (as of the annual report 2015). These counts cover four norovirus seasons, from 2011-W27 to 2015-W26, and are stratified by the 12 city districts and six age groups: 0–4, 5–14, 15–24, 25–44, 45–64, and 65+ years of age. The age groups were condensed from 5-year intervals to reflect distinct social mixing of pre-school vs. school children, and intergenerational mixing. Similarly stratified population numbers were obtained from the Statistical Information System Berlin-Brandenburg *StatIS-BBB* (http://www.statistik-berlin-brandenburg.de/statis) at the reference date December 31, 2011, when Berlin had 3 501 872 inhabitants in total.

**Fig. 1.**
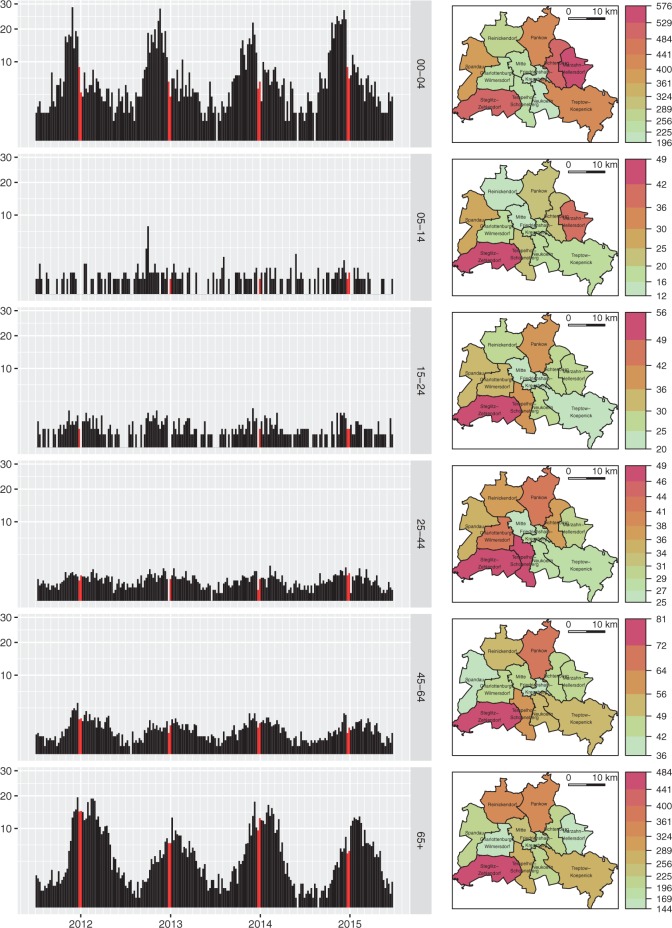
Age-stratified time series and maps of norovirus gastroenteritis incidence (per 100 000 inhabitants) in Berlin, 2011-W27 to 2015-W26. The weekly incidence plots on the left all use the same $\sqrt{}$-scale. The Christmas break in calendar weeks 52 and 1 is highlighted. The group-specific maps on the right show the mean yearly incidence by city district.

Figure 1 (left) shows the weekly norovirus incidence stratified by age group and aggregated over all city districts. The reported incidence is higher in pre-school children and the retired population than in the other age groups. The yearly seasonal pattern, with overall counts ranging from 7 to 214 cases per week, is approximately constant during the four years (supplementary Figure S1, See supplementary material available at *Biostatistics* online). The typical bump during the Christmas break could be related to reporting deficiencies and school closure (Hens *and others*, 2009). The time series of the 5- to 14-year-old children contains an outbreak caused by contaminated frozen strawberries, which were delivered almost exclusively to schools and childcare facilities (Bernard *and others*, 2014a). Comparing seasonality between the age groups, the peak incidence in pre-school children seems to precede the peak in the highest age group. Our age-structured modeling approach will help to address the question raised by Bernard *and others* (2014b), “whether this reflects a pattern of disease transmission from young to old in the community”—taking the spatial aspect of disease spread into account.

How disease incidence varies across the 12 city districts of Berlin is shown in Figure 1 (right). The south-western district Steglitz-Zehlendorf tends to be affected more and the central districts tend to be affected less than the remaining districts. This pattern is roughly consistent across age groups. An exception are the two younger age groups, which exhibit a relatively high incidence in Marzahn-Hellersdorf. District-specific seasonal shifts are not apparent (supplementary Figure S2, See supplementary material available at *Biostatistics* online).

Animated, age-stratified maps of the weekly counts encompass the full information from all three data dimensions. Such an animation (supplementary material) may provide additional insight into the dynamics of disease spread. However, epidemic models estimated from these data offer a more structured view and take population heterogeneity directly into account.

### 2.3. Contact data

**Fig. 2.**
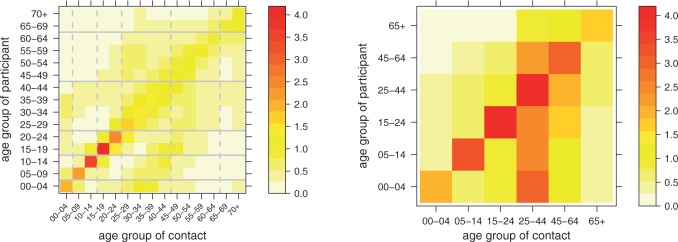
Age-structured contact matrix estimated from the German POLYMOD sample using 5-year intervals (left), and aggregated to the age groups of the surveillance data (right). The entries refer to the mean number of contact persons per participant per day.

We use contact data from the German subset of the POLYMOD study (Mossong *and others*, 2008), where both physical and non-physical (conversational) contacts have been recorded. We will report results based on all contacts and on physical contacts only. The age-structured social contact matrix $\boldsymbol{C} = (c_{g'g})$ contains the mean numbers of contact persons in age group $g$ during one day reported by a participant in age group $g'$. Instead of using sample means, we estimate $\boldsymbol{C}$ by the approach of Wallinga *and others* (2006), which accounts for the reciprocal nature of contacts. Each entry $c_{g'g}$ is assumed to be the mean of a negative binomial distribution, under the restriction $c_{g'g} n_{g'} = c_{gg'} n_{g}$, where $n_g$ is Berlin’s population in age group $g$. We estimate a detailed contact matrix with 5-year intervals, which we subsequently aggregate to the above six age groups (Figure 2). Direct estimation of the aggregated contact matrix leads to similar numbers.

The strong diagonal pattern in the social contact matrix reflects that people tend to mix with people of the same age. The other prominent pattern is produced by the contacts between parents and children. The matrix for physical contacts shows similar patterns (supplementary Figure S3, See supplementary material available at *Biostatistics* online). Aggregation of the contact matrix is done by summing over the contact groups (columns) to be joined and calculating the weighted average across the corresponding participant groups (rows), with weights equal to the group sizes. The aggregated contact matrix is asymmetric because of the different sizes of the involved age groups, but reciprocity at the population level still holds. For the models described in the next section, only the row-wise distributions will be relevant, i.e. the contact pattern of an infectious participant across the different age groups.

## 3. AN AGE-STRUCTURED SPATIO-TEMPORAL MODEL FOR INFECTIOUS DISEASE COUNTS

We review an endemic–epidemic modeling framework for areal time series of infectious disease counts (Meyer and Held, 2014, Section 3), into which we subsequently incorporate an additional stratification variable featuring a contact matrix.

### 3.1. Spatio-temporal formulation

Conditionally on past observations, the number of reported infections in region $r$ and time period $t$, $Y_{rt}$, is assumed to follow a negative binomial distribution with mean $\mu_{rt}$ and region-specific overdispersion parameters $\psi_r$ such that the conditional variance of $Y_{rt}$ is $\mu_{rt} (1+\psi_r \mu_{rt})$. The lower bound $\psi_r = 0$ yields the Poisson distribution as a special case, and a common simplifying assumption is that $\psi_r = \psi$ is shared across regions. In its most general formulation, the mean $\mu_{rt}$ is additively decomposed into *endemic* and observation-driven *epidemic* components as

(3.1) $$\begin{array}{cc} \mu_{rt} & \;=e_{rt}\;\nu_{rt}+\lambda_{rt}\;Y_{r,t-1}+\phi_{rt}\sum_{r^{\prime }\neq r}\left\lfloor w_{r^{\prime }r}\right\rfloor \;Y_{r^{\prime },t-1} \end{array}$$

with log-linear predictors

(3.2) $$\begin{array}{c} \begin{array}{cc} \log(\nu_{rt}) & \;=\alpha_{r}^{\left(\nu \right)}+{\beta^{\left(\nu \right)}}^{⊤}z_{rt}^{\left(\nu \right)}\;,\\ \log(\lambda_{rt}) & \;=\alpha_{r}^{\left(\lambda \right)}+{\beta^{\left(\lambda \right)}}^{⊤}z_{rt}^{\left(\lambda \right)}\;,\\ \log(\phi_{rt}) & \;=\alpha_{r}^{\left(\phi \right)}+{\beta^{\left(\phi \right)}}^{⊤}z_{rt}^{\left(\phi \right)}\;, \end{array} \end{array}$$

and normalized transmission weights $\left\lfloor {{w_{r'r}}} \right\rfloor := w_{r'r} / \sum_j w_{r'j}$, $w_{rr} = 0$. The regression terms in (3.2) often include sine–cosine effects of time to reflect seasonally varying incidence (Held and Paul, 2012), but $\boldsymbol{z}_{rt}^{(\cdot)}$ may also involve other explanatory variables, such as vaccination coverage (Herzog *and others*, 2011). The first, endemic component in (3.1) is typically modeled proportional to a (population) offset $e_{rt}$, and partially captures infections not directly linked to observed cases from the previous time period, e.g. due to traveling outside the study region (edge effects). The epidemic component splits up into autoregressive effects, i.e. reproduction of the disease within region $r$, and neighborhood effects, i.e. transmission from other regions $r'$. It has proven useful to account for population size also in $\log(\phi_{rt}) = \alpha_r^{(\phi)} + \tau \log(e_{rt})$, such that $\tau$ determines how “attraction” to a region scales with population size (Xia *and others*, 2004). Furthermore, transmission weights $w_{r'r}$ reflect the flow of infections from region $r'$ to region $r$. These weights may be based on additional movement network data (Paul *and others*, 2008; Schrödle *and others*, 2012; Geilhufe *and others*, 2014), but may also be estimated from the data at hand. A suitable parametric model is a power-law distance decay $w_{r'r} = o_{r'r}^{-\rho}$ in terms of the adjacency order $o_{r'r}$ in the neighborhood graph of the regions (Meyer and Held, 2014).

Estimating separate dynamics for the reproduction of the disease within a region on the one hand, and transmission from other regions on the other hand, goes back to the original model formulation of Held *and others* (2005), where only first-order neighbors have been incorporated. The parametric distance weights offer an appealing alternative to reflect predominant local autoregression in a simpler model with a single epidemic component:

(3.3) $$\mu_{rt}=e_{rt}\;\nu_{rt}+\phi_{rt}\sum_{r^{\prime }}\left\lfloor w_{r^{\prime }r}\right\rfloor \;Y_{r^{\prime },t-1}\;,$$

where the choice $w_{r'r} = (o_{r'r} + 1)^{-\rho}$ gives unit weight to local transmission ($r'=r$) and then decays as a power law in terms of adjacency order. With such a power law and the suggested population dependence of $\phi_{rt}$, the epidemic component of (3.3) constitutes a so-called gravity model (Xia *and others*, 2004; Höhle, 2016). Furthermore, this formulation uses fewer parameters and extends more naturally to an additional stratification variable.

### 3.2. Extension for stratified areal count time series

Extending the above spatio-temporal model to fit multivariate time series of counts $Y_{grt}$ stratified by (age) group in addition to region enables us to relax the simple assumption of homogeneous mixing within each region. More complex strata such as the interaction of age group and gender are equally possible and can be subsumed in the single group index $g$.

We assume that a contact matrix $\boldsymbol{C} = (c_{g'g})$ is given, where each entry $c_{g'g} \ge 0$ quantifies the average number of contacts of an individual of group $g'$ with individuals of group $g$. The spatio-temporal model (3.3) then extends to a three-dimensional version as

(3.4) $$\mu_{grt}=e_{grt}\;\nu_{grt}+\phi_{grt}\sum_{g^{\prime },r^{\prime }}\left\lfloor c_{g^{\prime }g}w_{r^{\prime }r}\right\rfloor \;\;Y_{g^{\prime },r^{\prime },t-1}\;,$$

where both the endemic and epidemic predictors may gain group-specific effects. How the counts from the previous period affect the current mean in group $g$ and region $r$ is now determined by a product of contact and spatial weights. The product ensures that cases from group $g'$ in region $r'$ are ignored if there are no contacts to group $g$ or if there is no flux of infections from region $r'$ to region $r$. The weights are row-normalized over all combinations of group and region: $\sum_{g,r} \left\lfloor {{c_{g'g} w_{r'r}}} \right\rfloor = 1$. Note that this normalization removes any differences in group-specific overall contact rates (the row sums of $\boldsymbol{C}$). Our model therefore does not distinguish between proportionate mixing, where the rows of the contact matrix only differ by a proportionality factor, and a matrix with identical rows. The weighted sum of past cases transmitted to group $g$ in region $r$ is scaled by $\phi_{grt}$. If $\phi_{grt} = \phi_g^{(G)} \phi_{r}^{(R)}$, the group-specific effects $\phi_g^{(G)}$ will adjust the columns of the contact matrix.

There are two special cases of the contact structure involved in the epidemic component. First, a contact matrix with identical rows implies that the mixing pattern of the $Y_{g',r',t-1}$ infectious cases does not depend on the group $g'$ they belong to. An example of such homogeneous mixing is a contact matrix where each row equals the vector of group sizes ($c_{g'g} = e_g$). If $\phi_{grt}$ contains group-specific effects, a simple matrix of ones ($\boldsymbol{C} = \bf{1}$) will induce the same contact structure. The other special case is a diagonal contact matrix $\boldsymbol{C} = \boldsymbol{I}$, which reflects complete absence of mixing. This is equivalent to formulating a separate spatio-temporal model (3.3) for each group. However, also in this case of no between-group mixing, the joint model formulation has the advantage of allowing for parsimonious decompositions of $\nu_{grt}$ and $\phi_{grt}$ into group and region effects. Borrowing strength across groups is especially useful in applications with low counts.

### 3.3. Parameterizing the contact matrix

Contact patterns derived from sociological studies might not fully match the characteristics of disease spread. For example, social networks are known to change during illness (van Kerckhove *and others*, 2013) and brief contacts are frequently not reported (Smieszek *and others*, 2014). We therefore suggest a parsimonious single-parameter approach to adaptively estimate the transmission weights as a function of the given contact matrix $\boldsymbol{C}$.

Our proposal is borrowed from Köchenhoff *and others* (2006), who progressively transform a misclassification matrix to establish an association between the amount of misclassification in a covariate and the corresponding parameter estimate. The proposed transformation is based on the eigendecomposition of the matrix $\boldsymbol{C}$ to raise it to the power of $\kappa \ge 0$,

(3.5) $$C^{\kappa }:=E\Lambda^{\kappa }E^{-1}\;,$$

where $\boldsymbol{\Lambda}$ is the diagonal matrix of eigenvalues and $\boldsymbol{E}$ is the corresponding matrix of eigenvectors. Translated to our setting, the parameter $\kappa$ measures the amount of transmission between the subgroups of the population. Specifically, $\kappa = 0$ corresponds to complete absence of between-group transmission ($\boldsymbol{C} = \boldsymbol{I}$), whereas $\kappa = 1$ leaves the contact matrix unchanged. If $\boldsymbol{C}$ is row-normalized, all rows of $\boldsymbol{C}^\kappa$ converge to the same distribution as $\kappa \to \infty$. The transmission pattern thus becomes independent of the group the infected individual belongs to. Because of this useful interpretation, we assume a pre-normalized contact matrix $\boldsymbol{C}$ in the remainder of this paper.

**Fig. 3.**
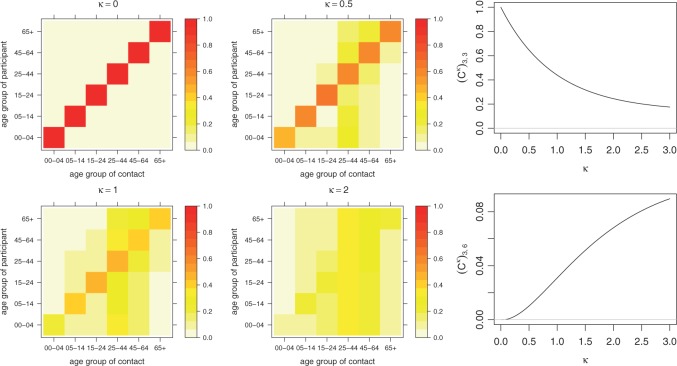
The power transformation $\boldsymbol{C}^\kappa$ (3.5) applied to the row-normalized POLYMOD contact matrix for different values of $\kappa$. The right-hand plots show the values of the diagonal entry $\lbrack 3,3\rbrack$ and the off-diagonal entry $\lbrack 3,6\rbrack$ of $\boldsymbol{C}^\kappa$.

The basic requirement that $\boldsymbol{C}$ can be factorized by an eigendecomposition will hold in most practical cases. However, we also need to make sure that $\boldsymbol{C}^\kappa$ has non-negative entries for $\kappa < 1$. With our contact matrix, two entries in $\boldsymbol{C}^\kappa$ become negative (but close to 0) for small $\kappa$. We follow a pragmatic approach and truncate negative entries at 0. Figure 3 exemplifies $\boldsymbol{C}^\kappa$ for the row-normalized version of the contact matrix from Figure 2, and illustrates how diagonal and off-diagonal entries, respectively, are affected by the power transformation.

### 3.4. Inference

Likelihood inference for the multivariate count time-series model (3.1) has been developed by Paul and Held (2011) and Meyer and Held (2014). The log-likelihood is maximized numerically using the quasi-Newton algorithm provided by the $\textsf{R}$ function $\texttt{nlminb}$ (R Core Team, 2016). Supplied with analytical formulae for the score function and Fisher information, convergence is fast, even for a large number of parameters. The modeling framework is implemented in the $\textsf{R}$ package $\texttt{surveillance}$ (Meyer *and others*, 2016, Section 5) as function $\texttt{hhh4}$.

The age-structured model (3.4) is built on top of the existing inference framework. The power parameter $\kappa$ of (3.5) is conveniently estimated via a profile likelihood approach (see, e.g. Held and Sabanés Bové, 2014, Section 5.3), which avoids the cumbersome implementation of additional derivatives with respect to all model parameters. We numerically maximize the log-likelihood of a model with fixed contact matrix $\boldsymbol{C}^\kappa$ as a function of $\kappa$. The profile confidence interval for $\kappa$ thus incorporates the uncertainty of all other parameter estimates (but not vice versa).

## 4. RESULTS

We apply an age-structured spatio-temporal model of the form (3.4) to the norovirus data described in Section 2. As the number of cases varies strongly by age group, we use group-specific overdispersion parameters $\psi_g$. For the mean, we assume the endemic–epidemic structure

(4.1) $$\begin{array}{cc} \mu_{grt} & \;=e_{gr}\;\exp\left\{\alpha_{g}^{\left(G\right)}+\alpha_{r}^{\left(R\right)}+\beta x_{t}+\gamma_{g}\sin(\omega t)+\delta_{g}\cos(\omega t)\right\}\\ & \;\;+e_{gr}^{\tau }\;\phi_{g}^{\left(G\right)}\phi_{r}^{\left(R\right)}\sum_{g^{\prime },r^{\prime }}\left\lfloor (C^{\kappa }{)}_{g^{\prime }g}\;(o_{r^{\prime }r}+1{)}^{-\rho }\right\rfloor \;Y_{g^{\prime },r^{\prime },t-1}\;. \end{array}$$

The endemic predictor allows for age- and district-specific incidence levels, fewer cases during the Christmas break ($x_t = 1$ in calendar weeks 52 and 1, otherwise $x_t=0$), as well as age-specific seasonality ($\omega = 2\pi/52$). Transmission between age groups is modeled using the power transformation (3.5) for the row-normalized contact matrix estimated from the POLYMOD study. Transmission between districts is quantified by a power law with respect to adjacency order. The intercepts are identifiable by fixing $\alpha_1^{(G)}=\alpha_1^{(R)}=0$, $\phi_1^{(G)}=\phi_1^{(R)}=1$, where $\phi_g^{(G)}$ and $\phi_r^{(R)}$ are estimated on the log-scale, and including overall intercepts in both model components.

Table 1 summarizes competing models with respect to the assumed contact structure between age groups. It turns out that a superposed epidemic component improves upon a purely endemic model, and that incorporating the contact matrix from the POLYMOD study outperforms naive models with homogeneous or no mixing between age groups. Akaike’s Information Criterion (AIC) is minimal for the model with a power-adjusted contact matrix $\boldsymbol{C}^\kappa$ (penultimate row), where the exponent is estimated to be $\hat{\kappa} =$ 0.47 (95% CI: 0.34–0.66). This means that the epidemic part subsumes more information from cases in the own age group than suggested by the original contact matrix (cf Figure 3). The change in AIC associated with this adjustment, however, is minor compared to the improvement achieved by employing the POLYMOD contact matrix in the first place. Results are very similar for *physical* contacts, but the fit is slightly worse.

**Table 1.** Model summaries for the age-stratified, areal surveillance data of norovirus gastroenteritis in Berlin. For reference, the first row represents the purely endemic model, which assumes independent counts. The remaining rows correspond to endemic–epidemic models with a spatial power law, but varying assumptions on the age-structured contact matrix $\boldsymbol{C}$. The columns refer to the following model characteristics: the number of parameters, the difference in AIC compared to the purely endemic model, the power $\tau$ of the population scaling factor, the decay parameter $\rho$ of the spatial power law, and the power adjustment $\kappa$ of the contact matrix. The parameter columns contain the estimates and 95% Wald confidence intervals

	dim	$\Delta$AIC	$\tau$	$\rho$	$\kappa$
Purely endemic model	36	0.0	—	—	—
Homogeneous mixing ($\boldsymbol{C} = \bf{1}$)	55	$-$415.4	1.19 (0.83–1.55)	2.43 (2.04–2.88)	—
No mixing ($\boldsymbol{C} = \boldsymbol{I}$)	55	$-$602.8	0.61 (0.24–0.98)	2.18 (1.89–2.53)	—
Original contact matrix $\boldsymbol{C}$	55	$-$631.9	0.97 (0.66–1.28)	2.34 (2.03–2.70)	—
Adjusted contact matrix $\boldsymbol{C}^\kappa$	56	$-$659.4	0.86 (0.53–1.19)	2.27 (1.98–2.61)	0.47 (0.34–0.66)
Based on physical contacts only	56	$-$655.3	0.85 (0.52–1.19)	2.27 (1.98–2.61)	0.48 (0.35–0.66)

The spatial spread of the disease across city districts is estimated to have a strong distance decay with $\hat{\rho} =$ 2.27 (95% CI: 1.98–2.61), such that the adjacency orders 0–4 have weights 1.00, 0.21, 0.08, 0.04, and 0.03. Supplementary Figure S4, See supplementary material available at *Biostatistics* online, shows age-dependent power laws (replacing $\rho$ by $\rho_{g'}$ in (4.1)), as well as unconstrained estimates of the order-specific weights, which are close to the power law. In accordance with the idea of a gravity model, we find that the epidemic part scales with the population size of the “importing” district and age group. Similar to a previous application on influenza (Meyer and Held, 2014), the corresponding estimate $\hat{\tau} =$ 0.86 (95% CI: 0.53–1.19) is slightly below unity and provides strong evidence for such an association.

**Fig. 4.**
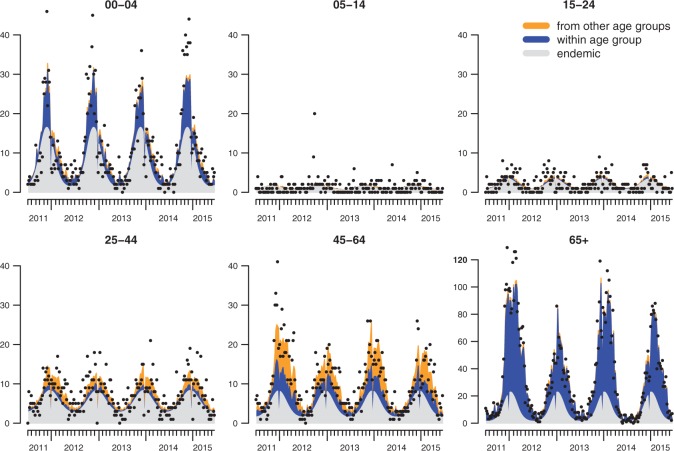
Fitted mean components from the AIC-optimal model with adjusted contact matrix, aggregated over all districts. The dots correspond to the reported numbers of cases.

Figure 4 shows the endemic–epidemic decomposition of the estimated mean aggregated across districts (supplementary Figures S5–S7, See supplementary material available at *Biostatistics* online for the district-level and overall fits). When reformulating the model as a multivariate branching process with immigration (Held and Paul, 2012), the largest eigenvalue of the matrix holding the estimated coefficients of $Y_{g',r',t-1}$ is 0.71, which can be interpreted as the overall epidemic proportion of disease incidence. However, this value mostly reflects the situation for the 65+ age group where the within-group spread is dominating. In contrast, for the groups of 5–14 and 15–24 year-old persons, almost no dependence on past counts of the same or the other age groups can be identified. Interestingly, the groups of 25–44 and 45–64 year-old persons seem to inherit a relevant proportion of cases from other age groups. The youngest age group, though, mostly depends on the endemic component and its own cases, which is probably related to their early onset. The age-dependent sine–cosine effects capture these shifts and are shown in supplementary Figure S8, See supplementary material available at *Biostatistics* online. The modal endemic incidence is in calendar weeks 48 (0–4), 45 (5–14), 52 (15–24), 51 (25–44), 52 (45–64), and 3 (65+), respectively. The largest amplitude is estimated for the youngest and oldest groups.

The estimated group-specific overdispersion parameters are 0.24 (0–4), 1.98 (5–14), 0.30 (15–24), 0.03 (25–44), 0.15 (45–64), and 0.40 (65+) in the model with adjusted contact matrix. The large overdispersion for the 5- to 14-year-old children may be partly due to the food-borne outbreak in 2012, for which the model does not explicitly account. The estimates are similar for the other epidemic models of Table 1, but slightly larger in the endemic-only model.

## 5. DISCUSSION

We have incorporated a social contact matrix in a regression-oriented, endemic–epidemic time-series model for stratified, area-level infectious disease counts. This three-dimensional approach provides a more detailed description of disease spread than unstratified or non-spatial models, which inherently assume homogeneous mixing within each region or subgroup, respectively.

In our application to age-stratified counts of norovirus gastroenteritis in Berlin’s city districts, the contact model was superior to homogeneous or no mixing between age groups. The model further improved when adjusting the POLYMOD contact matrix toward more within-group transmission. This could be related to biases in contact reporting (Smieszek *and others*, 2014) with more unreported (short) contacts along the diagonal. The two age groups involving parents were affected the most by preceding infections in other age groups. This is in accordance with the leading role of school children in influenza epidemics (Worby *and others*, 2015).

Furthermore, new infections predominantly depend on past cases from the same district, as suggested by the estimated spatial transmission weights. An age-dependent distance decay could not be identified from the disease counts. One could thus try to replace the parametric formulation by a social contact matrix, stratified by spatial distance in addition to age group. Separate movement data for school children and adults could then be used to quantify the strength of epidemiological coupling between regions (Kucharski *and others*, 2015). However, integration of movement network data does not necessarily improve predictions (Geilhufe *and others*, 2014).

A potentially more severe simplification of our model is the assumption of a time-constant contact matrix. Although weekday vs. weekend differences in contact patterns are not relevant for weekly time-series models, there are possibly relevant seasonal effects on larger time scales. For instance, the contact structure of school children changes considerably between regular and school holiday periods (Hens *and others*, 2009). Our model could be further tuned both by incorporating a time-varying contact matrix and by estimating seasonality also in the epidemic component (Held and Paul, 2012), which the $\texttt{hhh4}$ implementation already supports.

To check the robustness of our results with respect to under-reporting, we re-estimated the models with age-specific multiplication factors applied to the reported numbers of cases. Roughly following Bernard *and others* (2014c, Table 1), we used factors of 1.5 (0–4), 2.5 (5–14), 3.0 (15–24), 3.0 (25–44), 2.5 (45–64), and 2.0 (65+), respectively. While the overdispersion increases, the parameters of the mean are close to the original fit and the epidemic proportion is similar (supplementary Figure S9, See supplementary material available at *Biostatistics* online). For small strata with a low number of cases, a drawback of this simple deterministic approach is that zero reported counts remain zero regardless of the amount of under-reporting. More sophisticated adjustments are currently being investigated within a Bayesian modeling framework. In principle, asymptomatic infections could be similarly accounted for as missing cases, but they seem to play a minor role in disease transmission (Sukhrie *and others*, 2012). One-week-ahead forecasts or long-term simulations of the number of (symptomatic) infections, however, are of particular relevance for public health planning. Whether the improved model with social contact data also leads to better predictions will be described elsewhere.

## Supplementary Material

Supplementary Data
