# Supplementary material for: Incorporating social contact data in spatio-temporal models for infectious disease spread
Source: Biostatistics. 2016 Dec 24;18(2):338–51. doi: 10.1093/biostatistics/kxw051 (PMC5379927; doi:10.1093/biostatistics/kxw051)
Supplement: Supplementary Data [file kxw051_Supp.zip › figures.pdf]

Incorporating social contact data in spatio-temporal models for infectious disease spread

Supplementary Materials

SEBASTIAN MEYER*, LEONHARD HELD

Epidemiology, Biostatistics and Prevention Institute, University of Zurich, Hirschengraben 84, CH-8001 Zürich, Switzerland

sebastian.meyer@uzh.ch

This document contains additional figures from our analysis of norovirus infections by age group in Berlin, 2011–2015. Further supplementary files contain an animation of the data and the R source package `hhh4contacts`, respectively.

*To whom correspondence should be addressed.

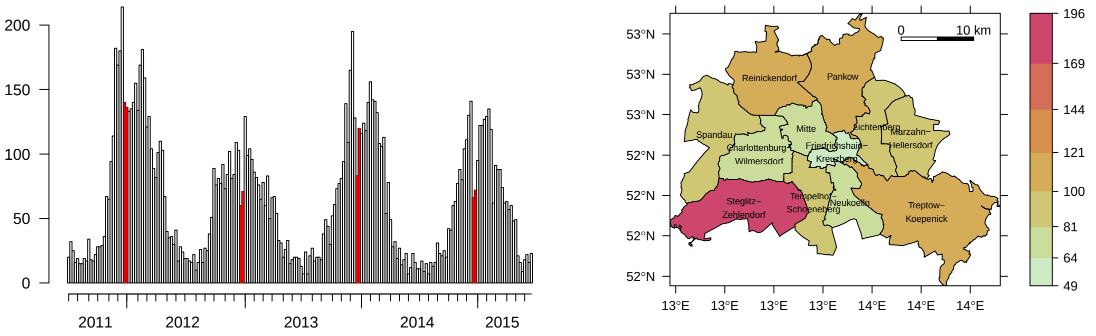

(a) Count time series. The yearly Christmas break in calendar weeks 52 and 1 is highlighted.

(b) Mean yearly incidence by city district (per 100 000 inhabitants).

Fig. S1: Norovirus gastroenteritis in Berlin, from 2011-W27 to 2015-W26.

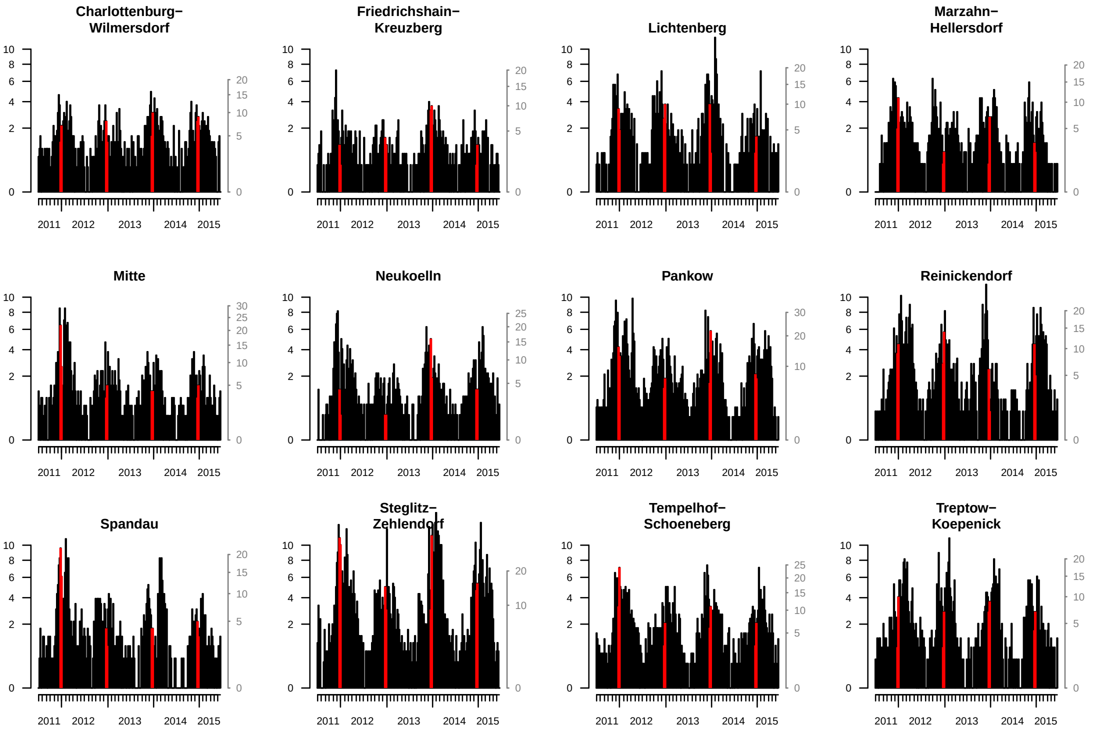

Fig. S2: Weekly norovirus gastroenteritis incidence (per 100 000 inhabitants) stratified by Berlin's 12 city districts. The incidence on the left axis obeys the same $\sqrt{}$-scale in all panels, while the corresponding counts can be read off from the right axis. The yearly Christmas break in calendar weeks 52 and 1 is highlighted.

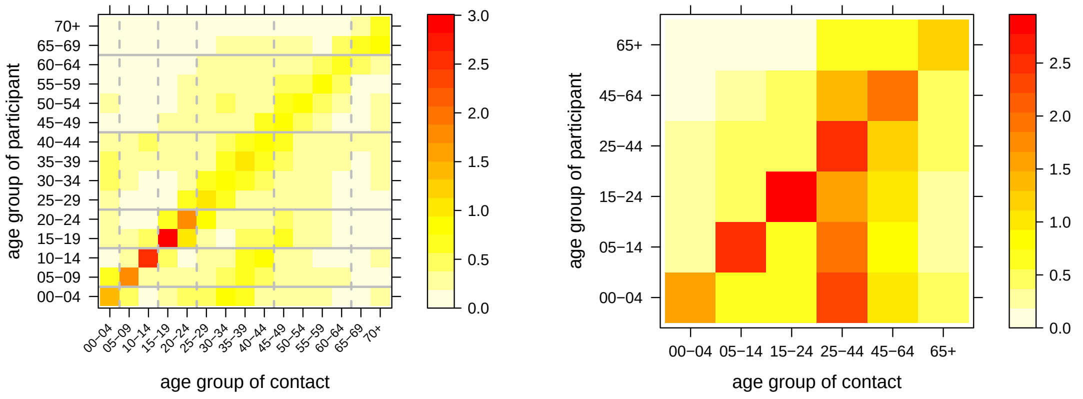

Fig. S3: Age-structured *physical* contact matrix estimated from the German POLYMOD sample using 5-year intervals (left), and aggregated to the age groups of the surveillance data (right). The entries refer to the mean number of persons contacted physically per participant per day.

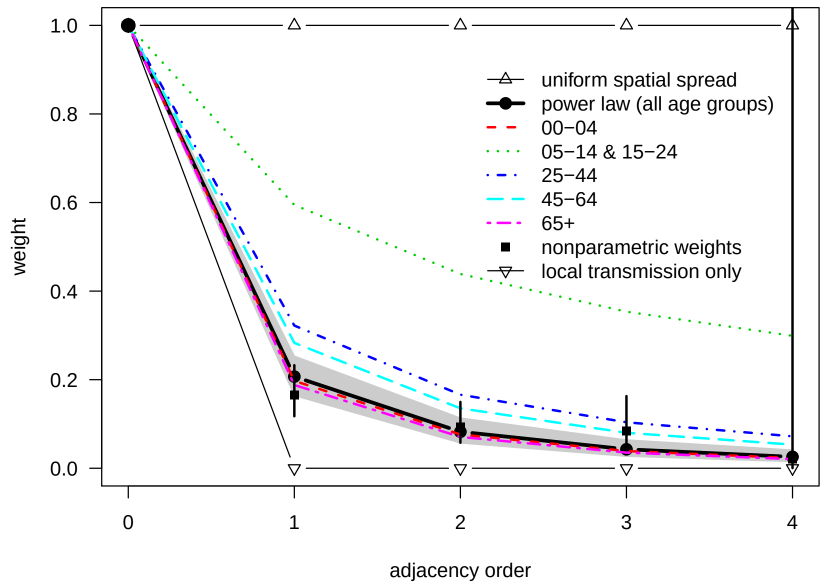

Fig. S4: Estimated (group-specific) power laws as well as unconstrained estimates of the spatial transmission weights $w_{r'r}$ as a function of adjacency order. The grey shading represents a 95% confidence interval for the joint power law, as do the vertical bars on top of the unconstrained estimates. For the low-incidence group of 5 to 14 year old children, a separate decay parameter was not identifiable. The shared power law with the 15 to 24 year old persons still has a large uncertainty. An alternative single-step kernel (not shown), which only discriminates within-district spread from homogeneous transmission to other districts, i.e. $w_{r'r} = \mathbb{1}(o_{r'r} = 0) + \omega \cdot \mathbb{1}(o_{r'r} > 0)$, provides a worse fit, where $\hat{\omega} = 0.11$ (95% CI: 0.08 to 0.15). We prefer the power-law model as a more sensible formulation, in particular for spatial extrapolation.

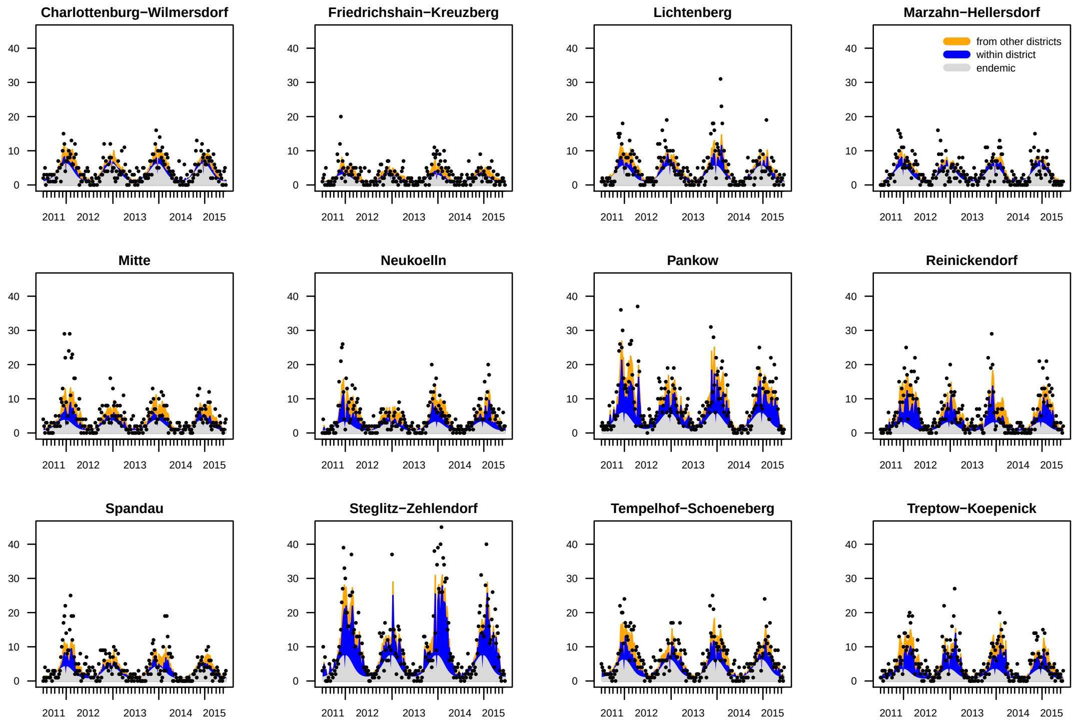

Fig. S5: Fitted mean components from the AIC-optimal model with adjusted contact matrix, aggregated over all age groups. The dots correspond to the reported numbers of cases.

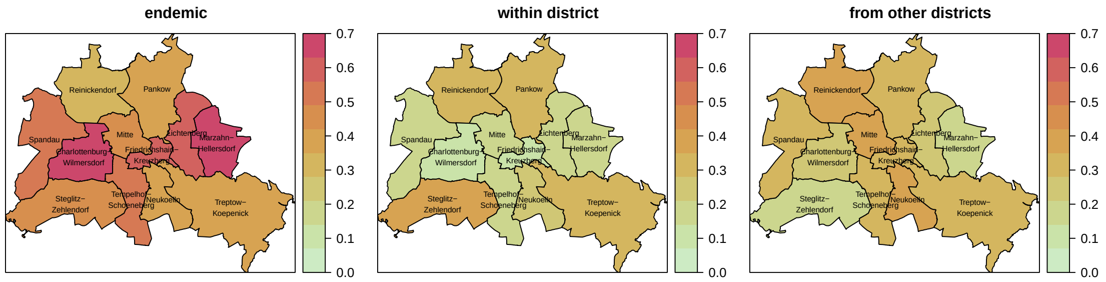

Fig. S6: Proportions of the district-specific mean attributed to various model components (for each district, the values of the three maps sum to 1). For this purpose, the fitted mean $\hat{\mu}_{grt}$ is aggregated over age groups and averaged over weeks.

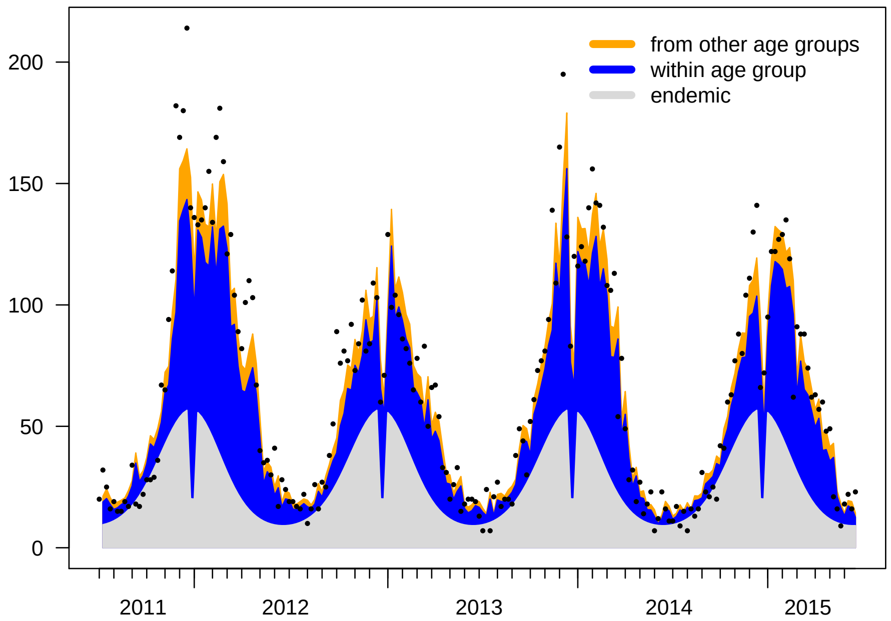

Fig. S7: Fitted mean components from the AIC-optimal model with adjusted contact matrix, aggregated over all districts and age groups. The dots correspond to the reported numbers of cases.

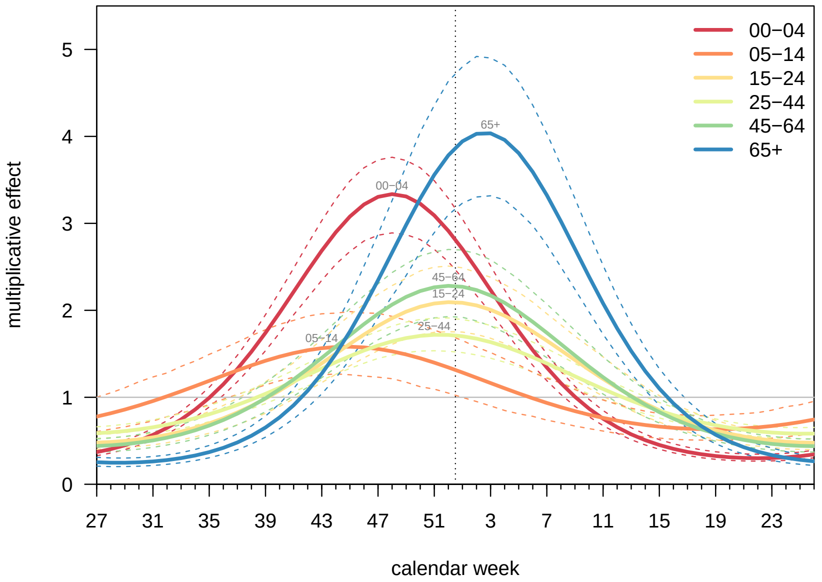

Fig. S8: Estimated age-dependent seasonality of the endemic model component with 95% pointwise confidence intervals.

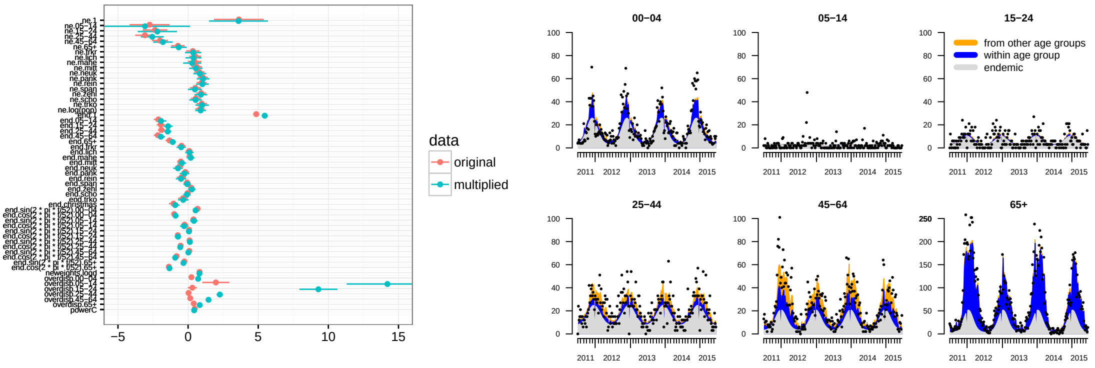

(a) Comparison of parameter estimates.

(b) Fitted mean components.

Fig. S9: AIC-optimal model fitted to counts multiplied by age-specific under-reporting factors.
